# Histologic Patterns of Connective Tissue Organization in the Subcrestal Peri-Implant Compartment: A Three-Case Report

**DOI:** 10.3390/jcm15207807

**Published:** 2026-10-10

**Authors:** Chiyun Won, Mi Heon Ryu

**Affiliations:** 1 Chiyun Won Dental Clinic, 2F, 46-1 Changjeon-ro, Mapo-gu, Seoul 04087, Republic of Korea; wonchiyun@gmail.com; 2 Department of Oral Pathology, School of Dentistry, Dental and Life Science Institute, Education and Research Team for Life Science on Dentistry, Pusan National University, Yangsan 50612, Republic of Korea

**Keywords:** dental implant, subcrestal implant placement, subcrestal zone, transitional zone, peri-implant soft tissue, connective tissue organization, histology, healing environment, case report

## Abstract

**Background**: Subcrestally placed implants (SPIs) may create a soft-tissue compartment between a subcrestal implant component and the adjacent crestal bone, but the histologic characteristics of this tissue remain incompletely understood. This report examined whether connective tissue retrieved from this environment exhibited identifiable patterns of organization compatible with the proposed Subcrestal Zone (SZ) framework. **Case Presentation**: Three human peri-implant soft-tissue specimens obtained during routine surgical or restorative procedures at SPI sites were examined using hematoxylin and eosin, Picrosirius red, and, where applicable, Masson’s trichrome staining. Case A demonstrated a circumferential ring-shaped connective tissue configuration. Case B showed apparent continuity between a coronal epithelial–connective tissue region compatible with the Transitional Zone and a deeper collagen-rich compartment compatible with the SZ. Case C demonstrated radial structural differentiation, with distinct collagen architectures on the metal-facing and bone-facing aspects. **Conclusions**: Organized collagen-rich connective tissue can be present within the subcrestal peri-implant environment. These observations are compatible with the proposed SZ framework but do not establish the SZ as a distinct tissue type or demonstrate its persistence, biologic function, or clinical benefit. Standardized comparative and longitudinal studies are required.

## 1. Introduction

Since the introduction of osseointegration, implant biology has been understood primarily through two interfaces: the osseointegrated bone-to-implant interface and the supracrestal epithelial–connective tissue barrier surrounding the transmucosal component [1,2,3,4,5,6,7,8,9,10,11]. These conventional models of supracrestal tissue attachment (STA) are particularly applicable to equicrestal implant placement, in which the transmucosal abutment–restoration complex is positioned at or above the crestal bone level. However, the widespread clinical use of subcrestally placed implants (SPIs) has introduced a biologic situation that is not fully explained by conventional STA models [12,13,14,15,16,17,18].

In SPIs, a portion of the abutment–restoration complex may remain below the crestal bone level while being surrounded by soft tissue rather than mineralized bone [12,13,14,15,16,17,18]. Within conventional peri-implant models, such a subcrestal soft-tissue interface may be interpreted as a remodeling region, an epithelialized peri-implant pocket, or a consequence of inflammation and marginal bone loss [19,20,21,22,23,24,25]. This interpretation reflects the broader tendency to understand peri-implant soft tissue primarily through periodontal and equicrestal implant paradigms.

Subcrestal placement is nevertheless widely used to accommodate crestal topography and facilitate an appropriate restorative emergence profile. In clinically stable SPIs, a thin soft-tissue interface may persist between the subcrestal abutment–restoration complex and adjacent crestal bone without progressive destructive bone change [12,15,16,17,18,19]. This raises a clinically relevant question: whether the tissue occupying this compartment necessarily represents pathologic pocket tissue or may, under selected conditions, exhibit organized connective tissue adaptation.

In the present framework, the term Subcrestal Zone (SZ) describes the soft-tissue compartment located between a subcrestal implant component and the adjacent crestal bone. Depending on the treatment stage, this component may be a submerged healing abutment, a transmucosal component, or a definitive restorative component. Earlier histologic observations of organized collagen fiber networks around implants support the possibility that this compartment may contain organized connective tissue under favorable conditions [26,27].

Within the broader Transitional Zone (TZ)–SZ framework, the TZ denotes the more coronal epithelial–connective tissue region associated with the transmucosal emergence pathway, whereas the SZ denotes the deeper connective tissue compartment adjacent to the abutment–restoration complex below the crestal bone level [19,26,27]. These regions may be spatially continuous rather than sharply separated. This framework may be relevant not only to intentionally deep implant placement but also to implants placed in sloped or irregular ridges, where partial subcrestal positioning may occur on one or more aspects of the implant platform.

Through three human histologic cases, the present study examines distinct patterns of connective tissue organization within the proposed SZ, including circumferential organization, vertical TZ–SZ continuity, and radial structural differentiation. Differences observed across closed and open healing environments are also described. The study is intended as a hypothesis-generating investigation and does not seek to establish the SZ as a distinct tissue type.

## 2. Materials and Methods

### 2.1. Study Design

This report presents three descriptive human histologic cases involving peri-implant soft tissue specimens associated with SPIs. The study was designed as a hypothesis-generating observational analysis rather than a quantitative comparative investigation.

### 2.2. Case Selection

The three cases were included because clinically indicated tissue removal during routine surgical or prosthetic treatment yielded peri-implant soft tissue specimens from clinically identified subcrestal compartments. In all cases, tissue removal was required because the tissue interfered with the seating of a surgical or prosthetic component; no peri-implant tissue was removed solely for research purposes. Accordingly, the specimens constituted clinically selected rather than randomly or protocol-driven samples.

Case A involved retrieval of a ring-shaped connective tissue specimen from the subcrestal peri-implant region during second-stage surgery. Case B involved partial retrieval of peri-implant soft tissue containing coronal and deeper connective tissue components because the narrow tissue passage prevented passive seating of the definitive restoration. Case C involved retrieval of circumferential tissue from the clinically identified subcrestal compartment because it interfered with abutment seating. The retrieved tissues were readily identified based on their subcrestal location and could be separated from the surrounding tissues along a discernible dissection plane.

Following clinically indicated retrieval, the specimens were oriented and processed to evaluate different aspects of peri-implant soft tissue architecture. Case A was processed to preserve its circumferential architecture around the subcrestal implant component, Case B to evaluate continuity between coronal and deeper soft tissue regions, and Case C to compare the metal-facing and bone-facing aspects of the tissue. Because specimen geometry, orientation, and sectioning strategy differed among the three cases, direct morphologic comparisons should be interpreted with caution.

### 2.3. Healing Environment Classification

The cases were also interpreted according to healing environment. A closed healing environment was defined as a situation in which the implant or subcrestal implant component was covered by primary mucosal closure during osseointegration, creating a relatively secluded subcrestal tissue environment with limited direct communication with the oral cavity during early healing. Cases A and C were classified as closed healing environments.

An open healing environment was defined as a situation in which a transmucosal component, such as a healing abutment, digital abutment, or definitive abutment, was connected during the osseointegration period, allowing the peri-implant soft tissue to heal in communication with the oral environment through the transmucosal passage. Case B was classified as an open transmucosal healing environment.

This classification was used descriptively to explore whether epithelial distribution, inflammatory cell localization, and collagen organization may differ according to the degree of direct oral communication during healing.

### 2.4. Histologic Examination

For histopathological examination, the retrieved soft tissues were fixed in formalin, processed using an automated tissue processor (Leica ASP 300S; Leica Biosystems, Deer Park, IL, USA), embedded in paraffin, and sectioned at a thickness of 4 μm. In Case A, the ring-shaped circumferential specimen was sectioned in a transverse plane perpendicular to the implant long axis to preserve and demonstrate its circumferential architecture. In Case B, the specimen containing both coronal and deeper tissue regions was sectioned in a longitudinal–radial plane parallel to the implant long axis, allowing evaluation of coronal–apical continuity between the TZ and SZ regions. In Case C, the cylindrically shaped circumferential tissue surrounding the implant abutment was longitudinally opened along a line parallel to the implant long axis and flattened before embedding. Sections were subsequently cut parallel to the broad surface of the flattened specimen. Accordingly, the histologic plane was oriented longitudinally and tangentially to the implant surface rather than perpendicular to the implant axis. The numbers of paraffin blocks processed were one for Case A, three for Case B, and two for Case C. From each paraffin block, one serial section was prepared for H&E staining and another serial section for Picrosirius red staining. For Case C only, an additional serial section from each of the two blocks was prepared for Masson’s trichrome staining. Accordingly, 2, 6, and 6 stained sections were evaluated for Cases A, B, and C, respectively.

Standard hematoxylin and eosin (H&E) staining was performed. Briefly, the tissue sections were deparaffinized in xylene and rehydrated through a graded ethanol series from 100% to 70%. The sections were stained with hematoxylin solution (Leica Biosystems) for 5 min, followed by eosin solution (Leica Biosystems) for 2 min. After dehydration through an ascending ethanol series (70%, 95%, and 100%; Duksan Pure Chemicals Co., Ltd., Ansan, Republic of Korea) and clearing in xylene, the sections were mounted using Permount™ Mounting Medium (Electron Microscopy Sciences, Hatfield, PA, USA).

For additional evaluation of collagen in Case C, Masson’s trichrome staining was performed on serial sections from the two paraffin blocks. The sections were deparaffinized, rehydrated, and stained with Weigert’s iron hematoxylin (BioGnost Ltd., Zagreb, Croatia) for 5 min. After washing, the sections were stained with Biebrich scarlet–acid fuchsin solution (Sigma-Aldrich, St. Louis, MO, USA) for 3 min, differentiated in a 3% phosphomolybdic–phosphotungstic acid solution (Polysciences, Inc., Warrington, PA, USA) for 2 min, and then stained with aniline blue (ScyTek Laboratories, Logan, UT, USA) for 5 min. The sections were treated with 1% acetic acid (Daejung Chemicals & Metals Co., Ltd., Siheung, Republic of Korea) for 1 min, dehydrated, cleared, and mounted using Permount™ Mounting Medium (Fisher Scientific, Waltham, MA, USA).

For Picrosirius red staining, serial sections from all paraffin blocks were deparaffinized, rehydrated, and stained with Weigert’s iron hematoxylin (BioGnost Ltd.) for 5 min. After washing, the sections were stained with Picrosirius red solution (Abcam, Cambridge, UK) for 60 min, followed by brief washing in two changes of 0.5% acetic acid solution (Daejung Chemicals & Metals Co., Ltd.). The sections were then dehydrated, cleared, and mounted using Permount™ Mounting Medium (Fisher Scientific).

All stained slides were digitized using a PANNORAMIC 250 Flash III slide scanner (3DHISTECH Ltd., Budapest, Hungary) with a 20× Plan-Apochromat objective and a 1.6× camera adapter for histopathologic evaluation. Images were acquired at a pixel resolution of 0.242797 μm/pixel in both the X and Y directions.

Evaluation of all stained slides was conducted using digitized whole-slide images. Picrosirius red-stained sections were evaluated using only the digitized images, without polarized-light microscopy. Histologic assessment focused on epithelial distribution, connective tissue organization, collagen density and architecture, inflammatory cell infiltration, spatial relationships between the coronal and deeper tissue regions, and radial structural differences between the metal-facing and bone-facing aspects where identifiable. All histologic evaluations were performed by an experienced oral pathologist (M. H. Ryu), who was aware of the case identity and specimen orientation; therefore, the assessment was not blinded.

This three-case report was prepared in accordance with the CARE guidelines. A completed CARE checklist is provided as Supplementary Material.

### 2.5. Case Presentations

#### 2.5.1. Case A

**Figure 1:**
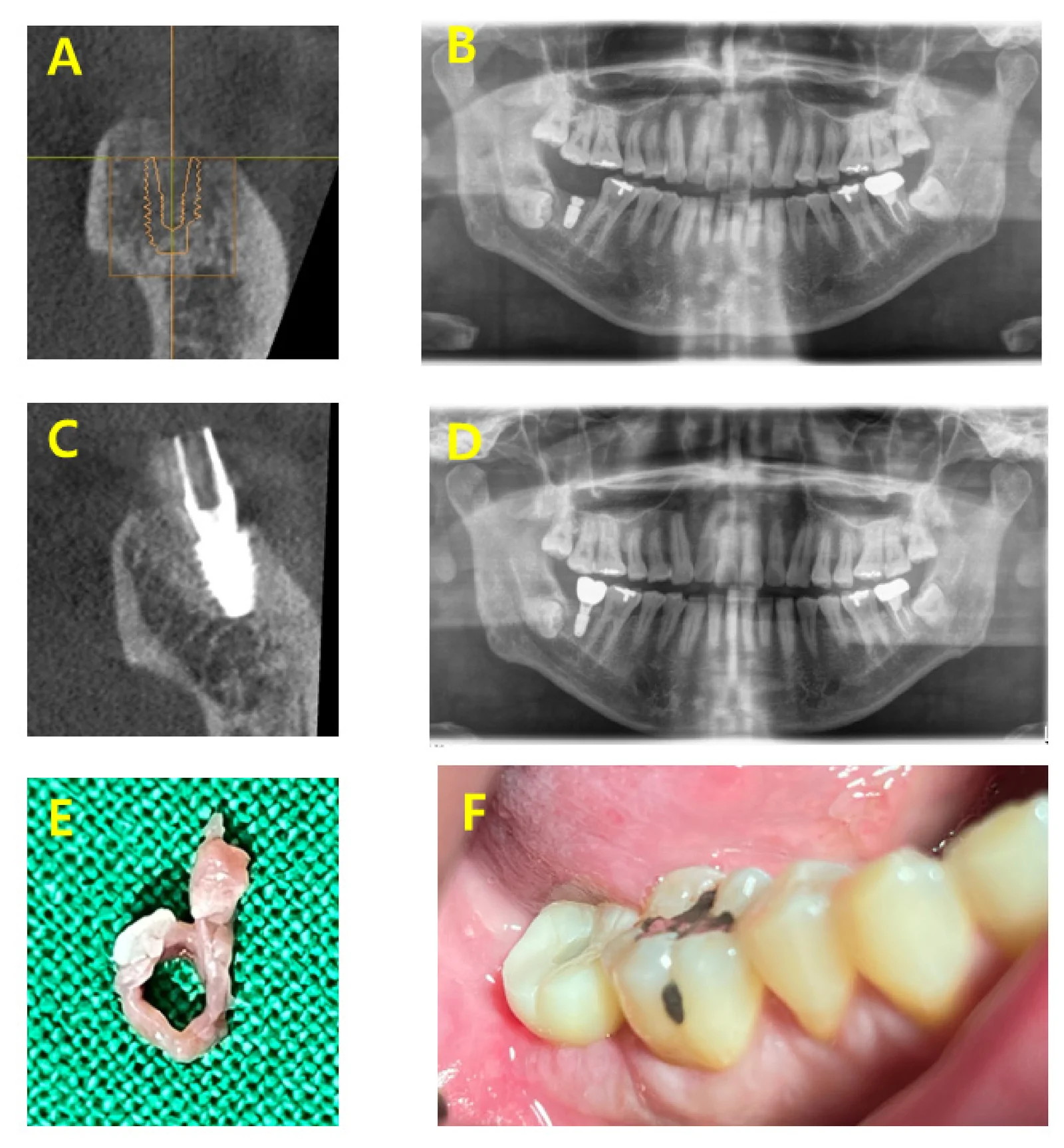
Clinical, radiographic, and gross specimen sequence of Case A. (**A**) Frontal cone-beam computed tomography (CBCT) view of the edentulous right mandibular second molar ridge before implant placement, demonstrating a buccolingual crestal slope. (**B**) Postoperative panoramic radiograph after subcrestal implant placement. A healing abutment measuring 4.8 mm in diameter and 2.0 mm in height was connected to the subcrestally positioned fixture and intentionally embedded to facilitate the subsequent second-stage procedure. The flap was closed for submerged healing during the osseointegration period. (**C**) Frontal CBCT view after connection of the definitive abutment. The definitive abutment measured 4.5 mm in diameter, with a 4.0 mm gingival cuff height and 5.5 mm abutment height. The gingival cuff portion was positioned within the subcrestal space, corresponding to the asymmetric implant position created by the sloped ridge morphology. (**D**) Panoramic radiograph after delivery of the implant-supported zirconia crown restoration. (**E**) Gross appearance of the retrieved ring-shaped soft tissue specimen obtained during the second-stage procedure. (**F**) Clinical appearance of the implant restoration, showing a maintained peri-implant soft-tissue contour without visible erythema or swelling and a favorable emergence profile.

A 48-year-old male non-smoker with a medical history of hypertension and hyperlipidemia, but no other relevant systemic conditions, presented with pain and mobility of the mandibular right second molar (FDI tooth 47). His general oral hygiene was considered satisfactory; however, the affected tooth exhibited severe site-specific periodontal involvement, including grade III mobility, a 10 mm buccal probing depth, and bleeding on probing. The clinical, radiographic, and specimen sequence for Case A is shown in Figure 1.

The tooth was extracted on 28 May 2025. Implant placement was subsequently performed on 29 December 2025, using an IS III implant (Neobiotech, Seoul, Republic of Korea; 5.0 mm diameter, 7.3 mm length). The interval between extraction and implant placement reflected the patient’s scheduling circumstances rather than a study-defined protocol.

Subcrestal placement was selected because the edentulous ridge exhibited a moderate buccolingual crestal slope. According to the SPI protocol applied in this case, the most apically positioned aspect of the crestal bone was used as the vertical reference, and the implant platform was positioned approximately 1.0 mm subcrestal to this reference. The vertical position was also selected to maintain at least 2.0 mm of lateral bone thickness around the implant fixture at the reference aspect. Because of the buccolingual crestal slope, the implant platform was consequently positioned more deeply relative to the other aspects of the ridge, reaching approximately 4.0 mm subcrestally on the lingual aspect. This protocol accommodated the three-dimensional ridge morphology without extensive crestal recontouring, thereby preserving the existing crestal bone. A healing abutment measuring 4.8 mm in diameter and 2.0 mm in height was connected and intentionally embedded in the subcrestal area. Primary closure was achieved, and healing was uneventful without wound dehiscence. This case was classified as a closed healing environment.

Second-stage surgery was performed on 24 March 2026. A definitive abutment was selected (4.5 mm diameter, 4.0 mm gingival cuff height, 5.5 mm abutment height). During abutment connection, removal of excess bone was required to avoid bone interference with proper abutment seating. This procedure was accompanied by removal of soft tissue corresponding to the subcrestal peri-implant region.

The operator directly identified the tissue within the subcrestal space between the embedded healing abutment and adjacent crestal bone. Its location and distinct separation plane allowed it to be distinguished from coronal transmucosal tissue. The tissue was dissected from this identified subcrestal region and retrieved as a continuous, intact ring-shaped specimen rather than as a fragment.

The specimen was fixed in formalin, embedded in paraffin, and sectioned in a transverse plane perpendicular to the implant long axis to preserve the ring-shaped architecture. The final zirconia restoration was delivered on 31 March 2026, using extraoral cementation. At the most recent follow-up on 12 August 2026, the implant remained in function, and clinical examination showed a maintained peri-implant soft-tissue contour without visible erythema, swelling, suppuration, or recession.

**Figure 2:**
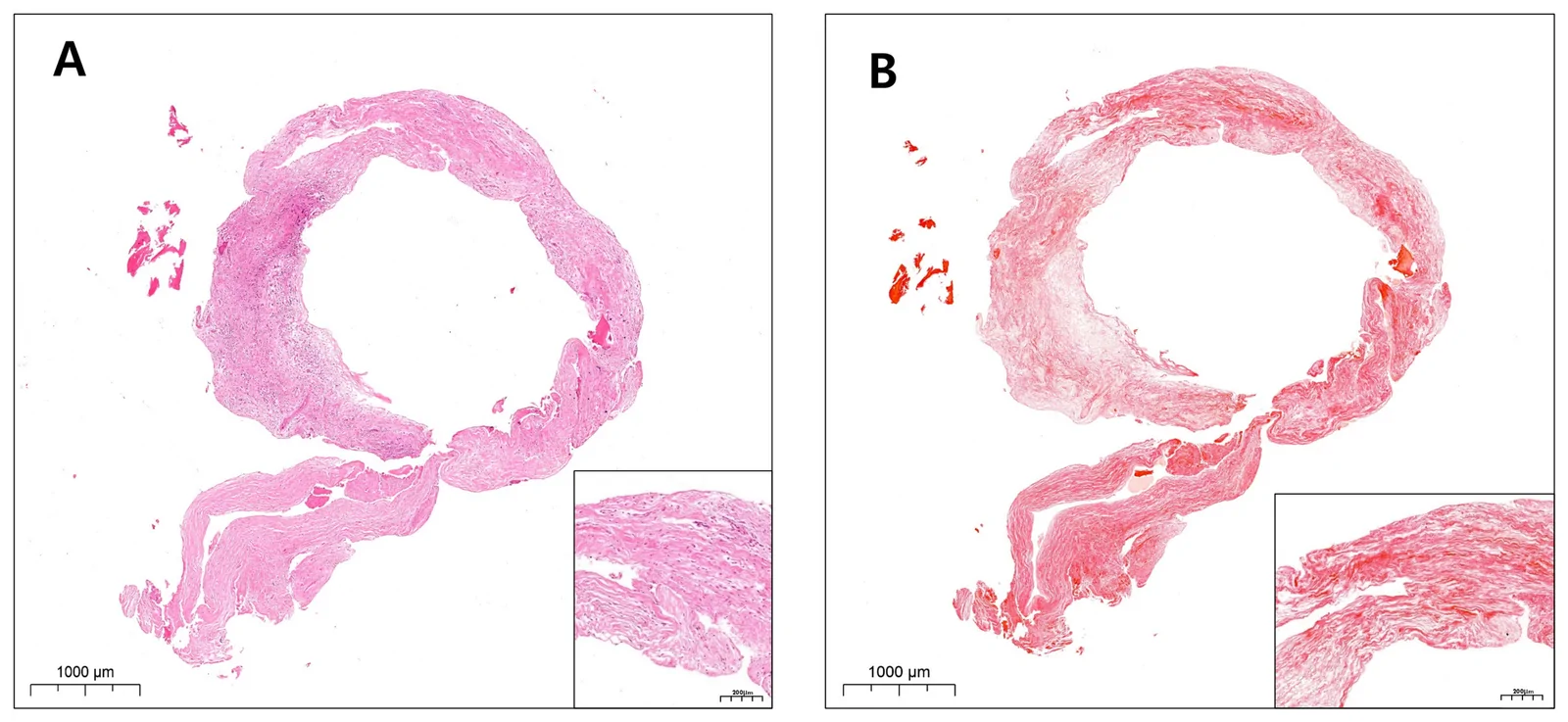
Histologic findings of the retrieved ring-shaped soft tissue specimen from Case A. (**A**) H&E staining showing a ring-shaped soft tissue specimen composed predominantly of dense fibrous connective tissue, with mild focal inflammatory infiltration and mild edema. The inflammatory cells were mainly lymphocytes with occasional plasma cells, suggesting a chronic inflammatory component. The tissue demonstrates circumferential architecture corresponding to the clinically retrieved subcrestal peri-implant soft tissue component (H&E, original magnification ×1.8; inset, ×11.6). (**B**) Picrosirius red staining of the same specimen, highlighting abundant collagen deposition throughout the ring-shaped tissue. Although focal areas of inflammation showed partial collagen disruption or localized collagen loss, dense collagen architecture was preserved in other regions of this clinically retrieved subcrestal specimen. However, these histologic findings alone do not establish the SZ as a distinct tissue type (Picrosirius red, original magnification ×1.8; inset, ×11.6).

The histologic findings of the retrieved Case A specimen are shown in Figure 2.

##### Histologic Findings of Case A

Histologic examination demonstrated a circumferential connective tissue structure composed predominantly of dense fibrous tissue. The overall architecture corresponded to the gross ring-shaped morphology observed during surgical retrieval.

The central empty space within the specimen was consistent with the previous position of the embedded healing abutment, suggesting that the tissue had formed around the subcrestal implant interface. No definite epithelial lining was identified in the deeper internal aspect of the examined specimen. Instead, the tissue demonstrated compact connective tissue architecture with variable collagen orientation.

H&E staining revealed dense fibrous connective tissue with relatively low cellularity in several regions, accompanied by localized mild inflammatory infiltration.

Picrosirius red staining demonstrated abundant collagen deposition throughout the specimen. The collagen architecture exhibited compact, regionally organized patterns, including partially circumferential and lamellar tendencies. Importantly, the specimen was not dominated by granulation tissue, extensive epithelial proliferation, or suppurative inflammatory change. These findings are compatible with an organized subcrestal connective tissue adaptation associated with an SPI.

#### 2.5.2. Case B

**Figure 3:**
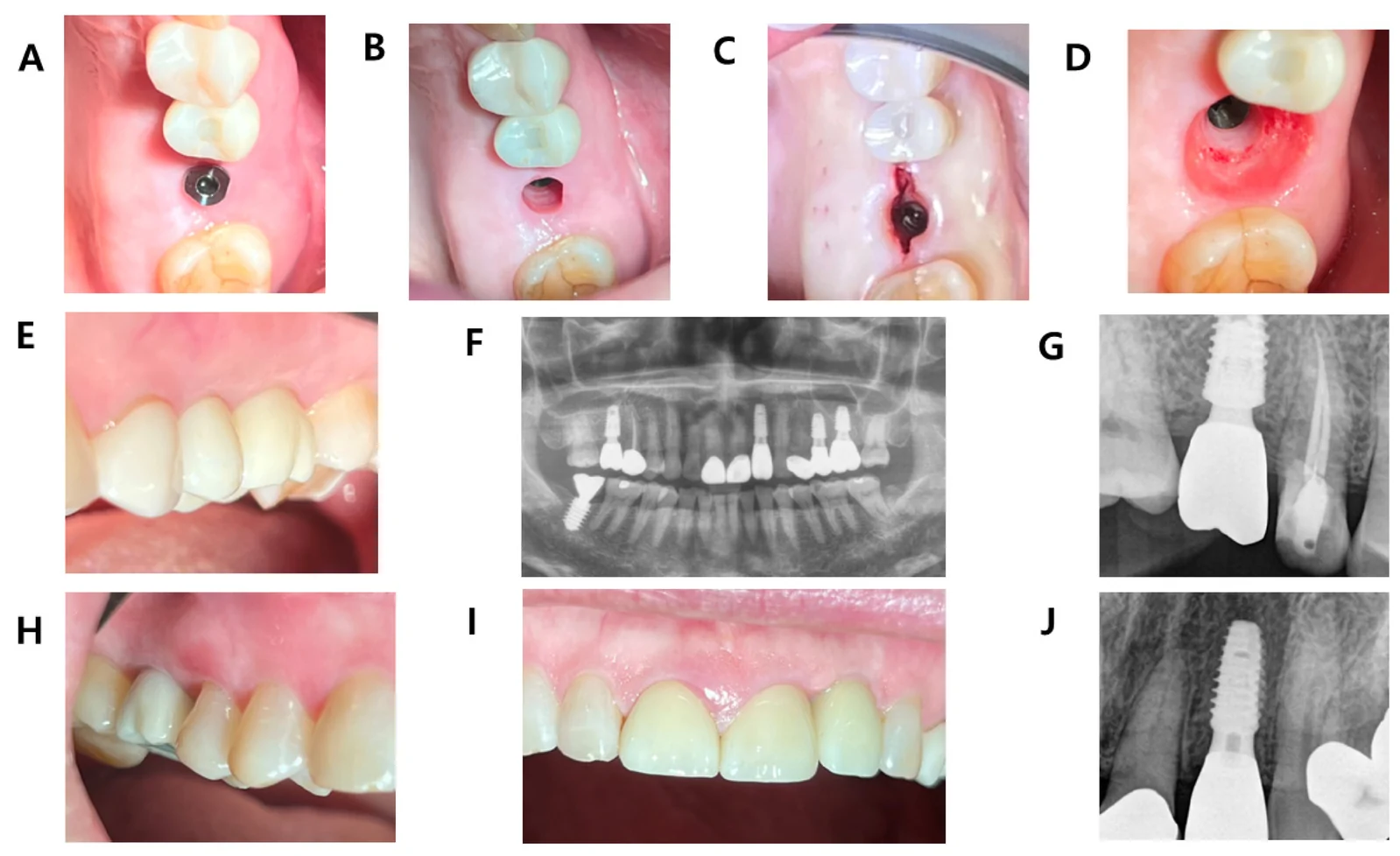
Clinical and radiographic sequence of Case B with same-patient long-term SPI observations. (**A**) Clinical view after implant placement with a digital abutment connected in the maxillary left first molar region. The digital abutment measured 5.0 mm in diameter with a 3.0 mm gingival cuff height and was maintained during the osseointegration period because adequate initial stability was achieved (>45 Ncm). The digital abutment also functioned as a scan body for intraoral scanning and fabrication of the final restoration. (**B**) Clinical view after removal of the digital abutment, showing a narrow transmucosal peri-implant soft tissue passage. Because the passage did not allow passive seating of the final restoration, soft tissue incision and partial excision were performed. The removed peri-implant tissue, clinically corresponding to the TZ/SZ region, was preserved for histologic examination. (**C**) Intraoperative occlusal view during soft tissue adjustment around the subcrestally placed implant. The peri-implant soft tissue was partially incised and excised to enlarge the transmucosal passage and accommodate the final restoration. (**D**) Clinical view of the enlarged transmucosal passage after adaptation to the final restoration. Gross inspection suggested regional color differences within the peri-implant soft tissue, including a pink oral sulcular epithelial region, a thicker reddish transitional region compatible with TZ, and a paler deeper region compatible with SZ. These visual differences suggested possible structural heterogeneity within the peri-implant soft tissue. (**E**) Final clinical appearance after delivery of the remade implant restoration, showing a maintained peri-implant soft-tissue contour without visible erythema or swelling, a favorable emergence profile, and esthetically harmonious integration with the adjacent dentition. (**F**) Panoramic radiograph obtained on 16 April 2026, showing the same patient’s maxillary implants, including the left first molar implant (Case B), the right first molar implant, and the left lateral incisor implant. This image provides an overview of the multiple SPI sites presented in this figure. The subcrestal region of the maxillary left first molar implant demonstrates close adaptation between the surrounding bone and the gingival collar portion of the abutment, corresponding clinically to the proposed SZ compartment. (**G**) Intraoral radiograph of the maxillary right first molar implant placed on 14 March 2019. This implant is presented solely as a long-term SPI observation from the same patient as Case B and was not the histologically sampled site. (**H**) Intraoral photograph showing a maintained peri-implant soft-tissue contour without visible erythema or swelling and a natural soft-tissue appearance around the maxillary right first molar implant shown in (**G**). (**I**) Intraoral photograph of the maxillary left lateral incisor implant placed on 27 February 2025, showing a maintained peri-implant soft-tissue contour without visible erythema or swelling and a natural soft-tissue appearance. This implant is presented as an additional long-term SPI observation from the same patient as Case B. (**J**) Intraoral radiograph of the maxillary left lateral incisor implant shown in (**I**). This implant is presented solely as an additional long-term SPI observation from the same patient as Case B and was not the histologically sampled site.

A 72-year-old female non-smoker with no relevant systemic conditions presented with a root fracture of the maxillary left first molar on 13 May 2025. Her general oral hygiene was considered satisfactory. The tooth was extracted on 22 May 2025, and socket preservation was performed using xenograft bone graft material. The clinical, radiographic, and specimen sequence for Case B is shown in Figure 3.

Implant placement was performed on 22 December 2025, using a Bright bone-level implant (Dentium, Seoul, Republic of Korea; 5.0 mm diameter, 7.0 mm length) with an internal conical connection and a platform-switched design. The fixture had a roughened surface except for the machined coronal shoulder/platform area. The initial stability was greater than 45 Ncm. A titanium digital abutment with a polished, machined transmucosal surface (5.0 mm diameter, 3.0 mm gingival cuff height) was connected at the time of implant placement to avoid a second-stage uncovering procedure and to facilitate the subsequent prosthodontic workflow. The surgical field was closed with the digital abutment in place. The digital abutment also served as a scan body for intraoral scanning and fabrication of the definitive restoration. Rather than reproducing the narrow contour of the 5.0 mm-diameter digital abutment, the restorative emergence profile was designed to approximate a natural tooth contour, using the dentogingival line of the adjacent teeth as a reference. Consequently, the definitive restoration had a broader and differently shaped emergence profile than the digital abutment used during healing. This case was classified as an open transmucosal healing environment.

Healing was uneventful. Because adequate primary stability (>45 Ncm) had been achieved, the 5.0 mm-diameter digital abutment was used as a transmucosal healing component during an approximately 3-month osseointegration period, thereby avoiding a second-stage uncovering procedure. The digital abutment also served as a scan body for intraoral scanning, allowing fabrication of the definitive restoration without a conventional impression procedure. During healing, however, the peri-implant soft tissue formed a narrow transmucosal passage corresponding to the contour and 5.0 mm diameter of the digital abutment rather than to the broader emergence profile intended for the definitive restoration. The definitive restoration had been designed to approximate a natural tooth contour using the dentogingival line of the adjacent teeth as a reference. Consequently, when delivery of the prefabricated definitive restoration was attempted on 16 March 2026, it could not be seated passively through the narrower soft-tissue passage. Clinically necessary incision and partial excision of the peri-implant soft tissue were therefore performed to enlarge the passage and accommodate the definitive restoration. During this procedure, the removed tissue, which included coronal and deeper regions corresponding clinically to the proposed TZ and SZ compartments, was retrieved and submitted for histologic examination.

Although the restoration was initially seated, a proximal contact discrepancy with the adjacent tooth was identified. A new restoration was fabricated after an additional impression procedure on 7 April 2026, and final delivery was performed on 28 April 2026. The definitive zirconia restoration was extraorally cemented to the abutment and delivered as a screw-retained restoration. Because cementation was performed extraorally and no cement was used intraorally, residual subgingival cement could be excluded as a potential contributor to the observed peri-implant tissue findings. The final clinical outcome was satisfactory in terms of appearance and proximal contact, and the patient did not experience food impaction. At the most recent follow-up on 29 September 2026, the implant remained in function, and clinical examination showed a maintained peri-implant soft-tissue contour without visible erythema, swelling, suppuration, or recession.

**Figure 4:**
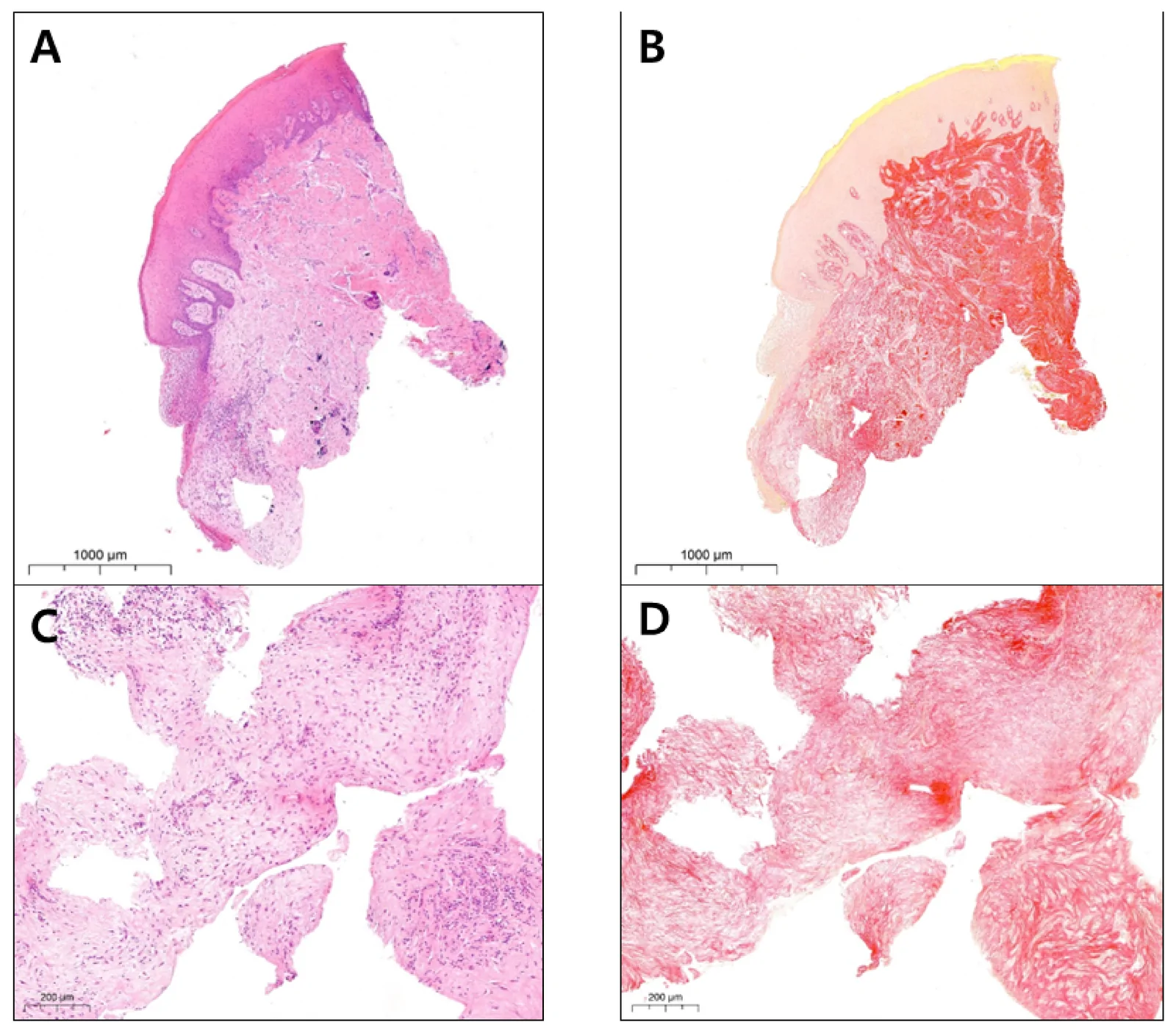
Histologic findings of Case B showing continuity between a coronal TZ-compatible region and a deeper SZ-compatible region, with higher-magnification views of the deeper tissue. (**A**) Low-magnification H&E staining of the mesial specimen showing a coronal epithelialized soft tissue component continuous with deeper fibrous connective tissue. The coronal tissue is compatible with the TZ, whereas the deeper collagen-rich connective tissue component is compatible with the SZ (H&E, original magnification ×4.9). (**B**) Low-magnification Picrosirius red staining of the mesial specimen highlighting collagen deposition extending from the coronal connective tissue region toward the deeper subcrestal tissue. The continuous collagen-rich architecture supports a structural transition from TZ-associated connective tissue to a deeper SZ-compatible compartment (Picrosirius red, original magnification ×5.1). (**C**) Higher-magnification H&E staining of the deeper subcrestal region showing fibrous connective tissue without a prominent epithelial lining. The tissue demonstrates scattered fibroblast-like cells, irregularly arranged collagen bundles, and mild focal inflammatory cell infiltration, consistent with a deeper SZ-compatible connective tissue component (H&E, original magnification ×8.0). (**D**) Higher-magnification Picrosirius red staining of the deeper subcrestal region demonstrating dense collagen organization corresponding to the H&E findings. These findings support the interpretation of the deeper tissue as a collagen-dominant SZ-compatible component (Picrosirius red, original magnification ×8.0).

The specimen from Case B was particularly informative because it contained both coronal and deeper peri-implant soft tissue components within a single specimen. This configuration allowed direct evaluation of spatial continuity between a coronal epithelial-connective tissue region compatible with the TZ and a deeper collagen-rich connective tissue compartment compatible with the SZ. Furthermore, because healing occurred around a transmucosal digital abutment, the case provided an opportunity to examine connective tissue organization within an open healing environment. The histologic findings of the retrieved Case B specimen are shown in Figure 4.

##### Histologic Findings of Case B

Histologic examination demonstrated clear regional variation between coronal epithelialized tissue and deeper collagen-rich connective tissue.

Low-magnification H&E staining showed stratified squamous epithelium with underlying fibrous connective tissue in the coronal region, consistent with a TZ-compatible epithelial–connective tissue interface. Importantly, the epithelialized coronal region was continuous with deeper connective tissue rather than sharply separated from it. No prominent epithelial lining was identified in the deeper tissue, which exhibited increased fibrous organization.

Picrosirius red staining demonstrated abundant collagen deposition extending from the subepithelial connective tissue into the deeper subcrestal region. Compared with the coronal portion, the deeper region demonstrated more compact and organized collagen architecture.

High-magnification examination of the deeper tissue demonstrated interwoven collagen bundles with scattered spindle-shaped fibroblast-like cells. Mild focal inflammatory infiltration was present, but the tissue was not dominated by granulation tissue or suppurative inflammatory destruction. No prominent epithelial lining was identified in the deeper examined fields.

These findings support the interpretation of the specimen as demonstrating vertical differentiation between a more coronal TZ-compatible epithelial-connective tissue component and a deeper collagen-rich connective tissue compartment compatible with SZ.

#### 2.5.3. Case C

**Figure 5:**
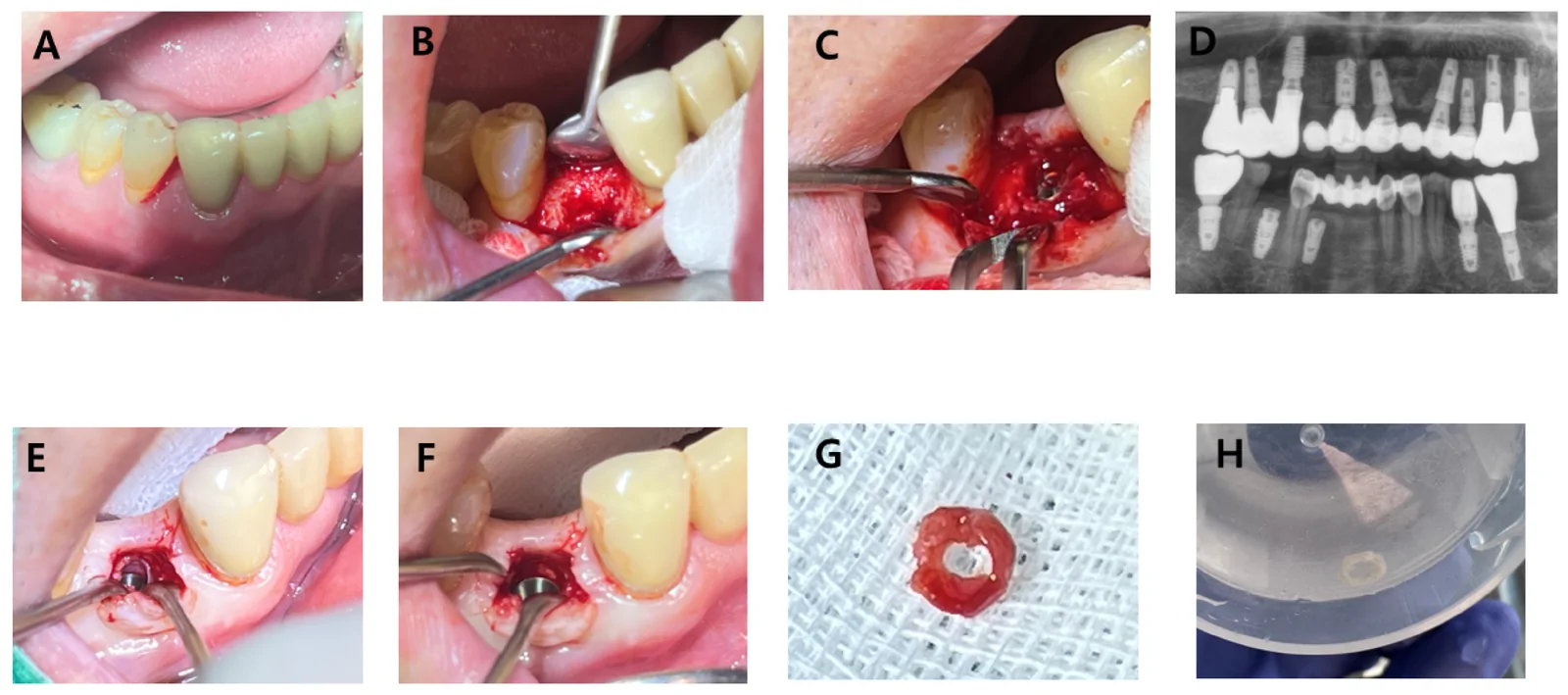
Clinical, radiographic, and gross specimen sequence of Case C. (**A**) Pre-extraction clinical view of the mandibular right first premolar region showing localized periodontal inflammation. (**B**) Intraoperative view after flap elevation at implant placement, demonstrating sloped crestal bone morphology. (**C**) Surgical view during implant placement showing subcrestal positioning selected to accommodate the crestal bone discrepancy. (**D**) Postoperative radiographic view confirming subcrestal implant placement. (**E**) Intraoperative view during second-stage surgery showing peri-implant soft tissue occupying the subcrestal interface. (**F**) Surgical field after gentle dissection and removal of the interfering circumferential subcrestal tissue, showing the crestal bone surface previously covered by the retrieved tissue and subsequent connection of the prosthetic component. The tissue was readily separated from the adjacent crestal bone and implant component during the clinically required procedure. (**G**) Gross appearance of the retrieved circumferential soft tissue specimen. (**H**) Retrieved specimen preserved for histologic examination.

A 72-year-old male non-smoker with a medical history of hypertension presented on 17 June 2025, with pain and mobility of the mandibular right first premolar (FDI tooth 44). His general oral hygiene was considered satisfactory. At the affected site, clinical examination revealed tooth mobility, localized gingival inflammation, and bleeding on probing. The clinical, radiographic, and specimen sequence for Case C is shown in Figure 5.

The tooth was extracted on 22 July 2025, followed by socket preservation. Implant placement was performed on 6 November 2025, using a Bright bone-level implant (Dentium, Korea; 4.5 mm diameter, 9.0 mm length) with an internal conical connection and a platform-switched design. Primary stability exceeded 30 Ncm. A healing cap measuring 3.8 mm in diameter and 1.5 mm in height was connected and completely submerged beneath the mucosa during closed healing. Intraoperative findings revealed a sloped crestal bone ridge. Given this anatomical discrepancy, subcrestal implant placement was selected to optimize adaptation to the crestal bone morphology. Postoperative radiographic evaluation showed subcrestal positioning of the implant. The surgical field was closed for submerged healing, and this case was classified as a closed healing environment.

At second-stage surgery on 15 January 2026, the operator directly observed circumferential soft tissue within the subcrestal space adjacent to the implant component and crestal bone. Its location and separation plane were identified during surgical exposure, allowing the tissue to be distinguished from the coronal mucosa. Because the tissue interfered with complete abutment seating, it was gently dissected and readily separated from the adjacent crestal bone and implant component, exposing the crestal bone surface that it had covered, and was retrieved as a continuous ring-shaped specimen. The intraoperative sequence and retrieved specimen are shown in Figure 5.

The specimen was immediately preserved in formalin for histologic analysis. Final restoration was delivered on 22 January 2026. At the most recent follow-up on 18 May 2026, the implant remained in function, and clinical examination showed a maintained peri-implant soft-tissue contour without visible erythema, swelling, suppuration, or recession.

Case C was selected because it provided a circumferential SZ-compatible specimen that allowed evaluation of radial structural differentiation between the metal-facing and bone-facing aspects of the subcrestal peri-implant tissue.

##### Histologic Findings of Case C

Histologic examination demonstrated dense fibrous connective tissue without a prominent epithelial component and with minimal inflammatory infiltration. The specimen showed abundant collagen-rich connective tissue, supporting compatibility with an organized subcrestal connective tissue compartment.

**Figure 6:**
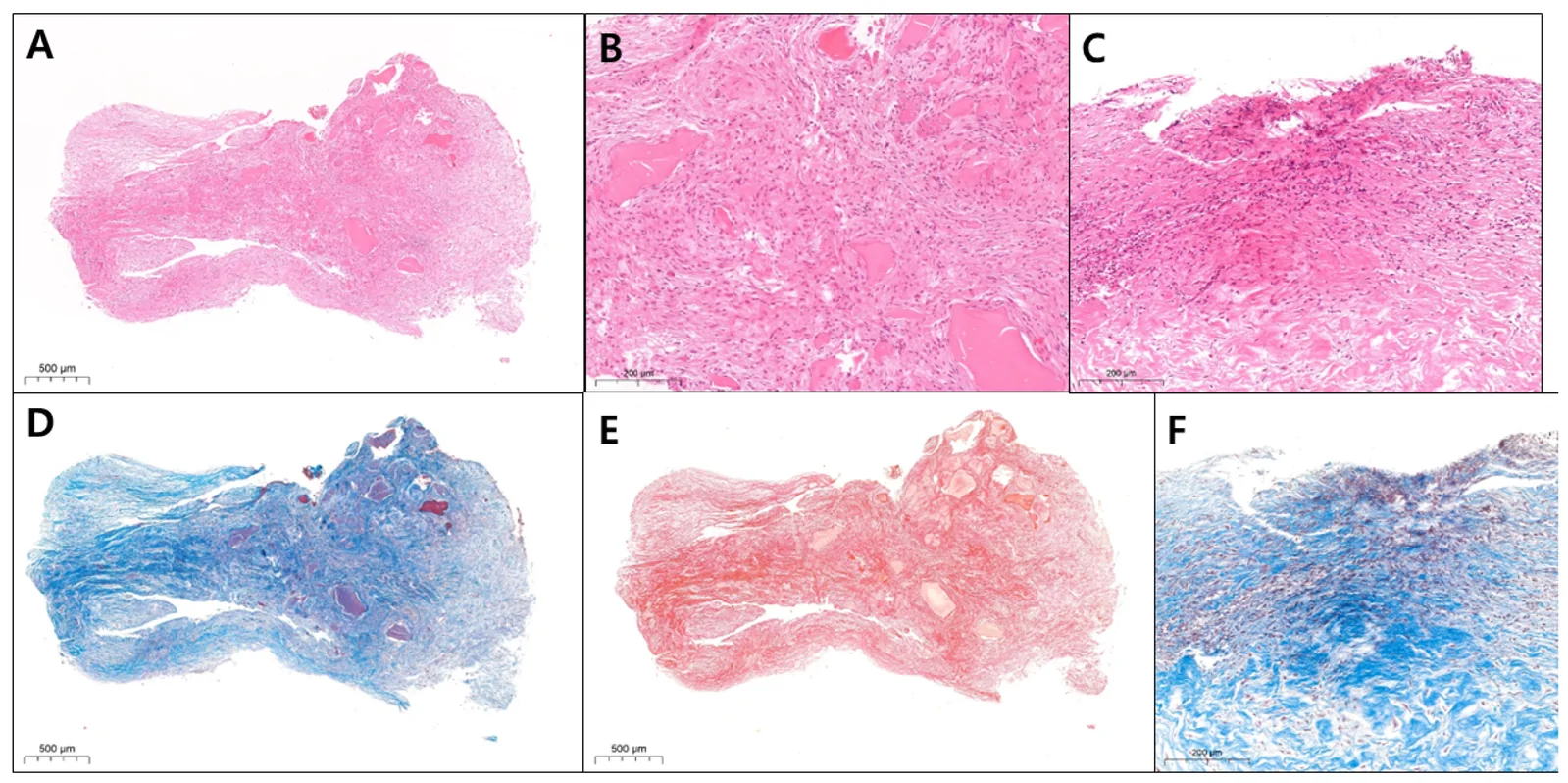
Histologic findings of Case C showing regional differences between the bone-facing and metal-facing aspects of an SZ-compatible specimen. (**A**) Low-magnification H&E staining showing dense collagen-rich connective tissue with focal osteoid-like or immature bone-like material and minimal inflammatory infiltration, retrieved from the subcrestal peri-implant region of the bone-facing aspect (H&E, original magnification ×2.8). (**B**) Higher-magnification H&E view of the bone-facing aspect showing interwoven, multidirectional collagen fibers associated with immature bone-like structures (H&E, original magnification ×11.5). (**C**) Higher-magnification H&E view of the metal-facing aspect showing compact and more parallel collagen bundles adjacent to the metal-facing surface (H&E, original magnification ×11.6). (**D**) Masson’s trichrome staining demonstrating well-organized collagen bundles within the retrieved specimen from the bone-facing aspect (Masson’s trichrome, original magnification ×3.0). (**E**) Picrosirius red staining highlighting collagen-rich architecture and regional differences in collagen fiber organization in the bone-facing aspect. The findings are broadly consistent with the connective tissue organization observed on Masson’s trichrome staining (Picrosirius red, original magnification ×3.0). (**F**) Higher-magnification Masson’s trichrome staining showing tightly arranged collagen bundles within the metal-facing aspect of the retrieved specimen (Masson’s trichrome, original magnification ×11.6).

A key finding in Case C was radial structural differentiation within the tissue. The bone-facing aspect showed interwoven and multidirectional collagen fibers, frequently associated with bone-like structures. This pattern was observed on the bone-facing aspect of the specimen. The histologic findings of the retrieved Case C specimen are shown in Figure 6.

In contrast, the metal-facing aspect demonstrated more compact, parallel collagen bundles, forming a dense, organized interface adjacent to the presumed implant-facing surface. The observed differences in collagen bundle arrangement between the bone-facing and metal-facing aspects indicate radial variation within this specimen. Whether these differences are related to the adjacent interfaces cannot be determined from a single specimen.

Taken together, the circumferential gross morphology, epithelial-poor connective tissue architecture, minimal inflammatory infiltration, and radial collagen organization support the interpretation of this specimen as a collagen-rich connective tissue specimen retrieved from the proposed subcrestal compartment associated with an SPI.

### 2.6. Histologic Observations

#### 2.6.1. Epithelial Distribution

The three cases demonstrated distinct epithelial patterns according to their clinical and spatial context. Cases A and C demonstrated predominantly connective tissue architecture without a clearly identifiable epithelial lining in the deeper internal regions of the circumferential specimens. These findings indicate that a clearly identifiable epithelial lining was not observed in the deeper regions of the examined specimens; however, this observation alone does not exclude inflammatory or pathological tissue characteristics.

Case B demonstrated a more coronal epithelialized component associated with stratified squamous epithelium and underlying connective tissue. However, the deeper portion of the same specimen demonstrated reduced epithelial involvement and increased connective tissue organization. Together, these findings suggest possible vertical differentiation between coronal peri-implant epithelial adaptation and deeper subcrestal connective tissue organization.

#### 2.6.2. Connective Tissue Organization

All three specimens demonstrated abundant connective tissue with organized collagen architecture. Case A demonstrated a circumferential connective tissue configuration with compact collagen organization throughout the specimen. Case B demonstrated continuity between coronal connective tissue and a deeper collagen-rich region with increased fibrous organization. Case C demonstrated radial structural differentiation, with different collagen patterns on the metal-facing and bone-facing aspects.

Picrosirius red staining highlighted dense collagen deposition in the examined specimens, particularly in the deeper subcrestal regions.

#### 2.6.3. Relationship Between the TZ and SZ

Case B provided the clearest evidence for possible continuity between TZ-compatible and SZ-compatible tissue regions. The coronal portion demonstrated epithelial-connective tissue characteristics compatible with conventional peri-implant soft tissue attachment, whereas the deeper region demonstrated a more collagen-rich and less epithelialized architecture.

Importantly, these regions appeared continuous rather than sharply separated, suggesting that the TZ and SZ may represent structurally differentiated but biologically related peri-implant soft-tissue regions.

#### 2.6.4. Radial Structural Differentiation Within the SZ

Case C provided evidence that SZ-compatible tissue may not only be circumferentially organized, but also radially differentiated. The metal-facing aspect demonstrated compact and more parallel collagen bundles, whereas the bone-facing aspect demonstrated more interwoven and multidirectional collagen architecture associated with bone-like structures. These findings describe radial differences in collagen organization within the Case C specimen; whether they are related to the adjacent interfaces cannot be determined from this single specimen.

#### 2.6.5. Closed and Open Healing Contexts

Cases A and C underwent closed healing, whereas Case B underwent open transmucosal healing around a digital abutment. In Case B, the single retrieved specimen contained both a coronal epithelialized region and a deeper collagen-rich region with less apparent epithelial involvement. This within-specimen observation provides an example of tissue organization in an open healing context. Whether open and closed healing conditions influence epithelial distribution or connective tissue organization remains a question for standardized comparative studies.

#### 2.6.6. Inflammatory Findings

Inflammatory infiltration was present in some regions but demonstrated variable distribution. Case A demonstrated mild localized inflammatory infiltration without dominance of granulation tissue or destructive inflammatory architecture. Case B demonstrated more apparent inflammatory infiltration in some coronal and transitional regions, whereas selected deeper regions retained compact connective tissue organization. Case C demonstrated minimal inflammatory infiltration in the examined SZ-compatible tissue.

These observations describe regional differences in inflammatory cell distribution within the examined sections. However, because the assessment was qualitative and based on H&E morphology alone, these findings neither establish that the subcrestal compartment is non-pathologic nor definitively distinguish it from peri-implantitis-associated tissue.

#### 2.6.7. Structural Diversity of SZ-Compatible Tissue

The three cases demonstrated different patterns of subcrestal connective tissue organization. Case A demonstrated a circumferential ring-shaped connective tissue configuration. Case B demonstrated vertically differentiated tissue continuity between coronal and deeper compartments. Case C demonstrated radial structural differentiation between metal-facing and bone-facing tissue aspects.

Importantly, all three specimens were obtained from tissue that had developed adjacent to healing or transmucosal components before definitive restoration and functional loading. Accordingly, the present histologic findings characterize healing-stage peri-implant tissues and should not be extrapolated to mature tissues surrounding definitive restorations under functional loading.

The observed structural diversity should be interpreted in the context of differences in implant position, local anatomy, prosthetic contour, healing environment, and specimen orientation.

## 3. Discussion

The present investigation arose from a recurring clinical observation: a soft-tissue space may remain between the subcrestal abutment–restoration complex and adjacent crestal bone. Although this space can be recognized clinically and radiographically, the tissue occupying it has received limited histologic interpretation. The present study therefore examined three peri-implant soft-tissue specimens retrieved from this subcrestal environment to determine whether they exhibited identifiable patterns of organization compatible with the proposed SZ framework.

Within conventional peri-implant pathology models, deep peri-implant soft tissue may be associated with tissue remodeling, epithelial migration, inflammatory destruction, and peri-implant pocket formation [20,21,22,23,24,25]. The subcrestal connective tissue specimens examined in the present study could therefore be interpreted as nonspecific fibrous or chronically inflamed tissue. Histopathologically, active or advanced peri-implantitis lesions often exhibit dense inflammatory cell infiltration, granulation tissue formation, vascular proliferation, and prominent collagen degradation [23,24,25]. In the present cases, however, the examined sections showed relatively organized collagen-rich connective tissue with localized or mild inflammatory cell infiltration, without dominant histologic features of tissue destruction.

Nevertheless, peri-implantitis-associated tissue may exhibit variable histopathologic features according to the stage and activity of the lesion. Because microbiologic profiling, immunohistochemical characterization using specific inflammatory cell markers, and direct comparison with confirmed peri-implantitis tissue were not performed, the present H&E-based observations remain qualitative and descriptive. They neither exclude inflammatory pathology nor independently establish peri-implant health. Future comparative studies incorporating quantitative histomorphometry, immunohistochemical analyses, and appropriate control tissues are required to clarify the inflammatory status and biological significance of subcrestal connective tissue. Moreover, the specimens in this study were collected during the initial healing phase, before definitive restoration and functional loading, rather than after long-term clinical function; this represents an important limitation of the study.

The biologic identity of the SZ-compatible tissue observed in the present cases remains uncertain. Cross-sectional histologic examination can characterize tissue architecture but cannot determine whether a connective tissue compartment represents a transient reactive response, a persistent adaptation, or a functionally specialized structure. Accordingly, the SZ should be interpreted in the present study as a spatially defined subcrestal compartment rather than as a distinct histologic tissue type. The tissue occupying this compartment may represent organized connective tissue adaptation, reactive tissue, or a combination of responses depending on local and healing conditions. The present findings do not establish long-term persistence, protective function, or an independent tissue identity. Comparative and longitudinal studies incorporating histomorphometric, immunohistochemical, and microbiologic analyses are required to determine its biologic identity and clinical significance.

**Figure 7:**
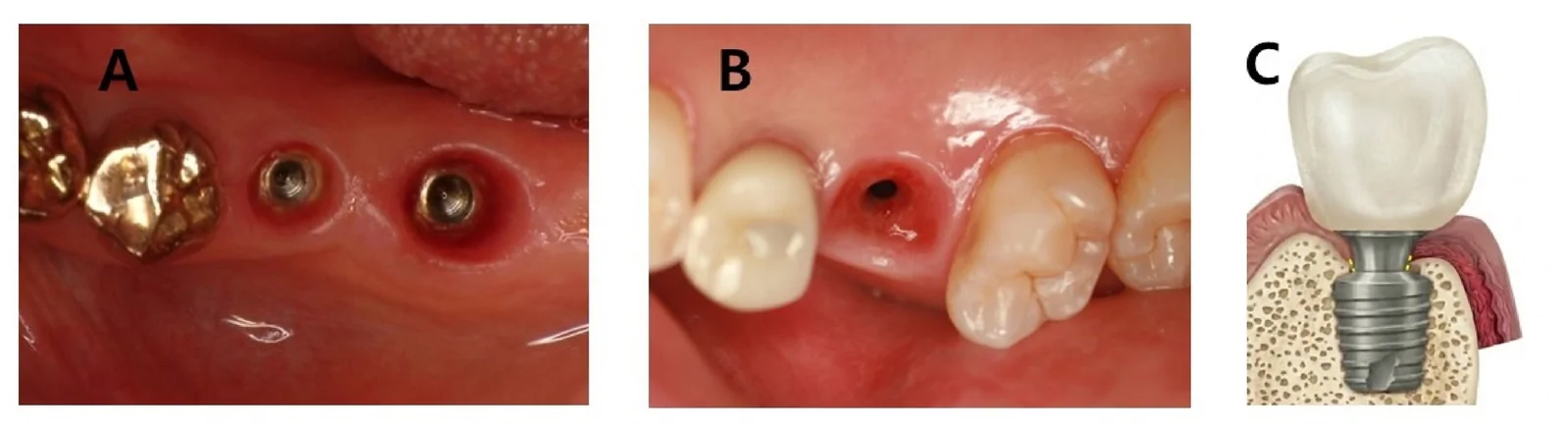
Clinical and conceptual comparison of the transmucosal peri-implant soft-tissue interface in equicrestal and subcrestal implant placement. (**A**) Clinical example of an equicrestally placed implant (EPI). Following removal of the healing component, the peri-implant soft-tissue passage appears relatively shallow and is largely confined to the supracrestal mucosal compartment. (**B**) Clinical example of a subcrestally placed implant (SPI). Following removal of the transmucosal component, the peri-implant soft-tissue passage forms a deeper funnel-shaped corridor extending below the crestal bone level. (**C**) Conceptual representation of the SPI configuration. Within the proposed TZ–SZ framework, the coronal portion of the transmucosal soft-tissue corridor corresponds predominantly to the Transitional Zone (TZ), whereas the deeper portion located between a subcrestal implant component and the adjacent crestal bone corresponds to the Subcrestal Zone (SZ). The dashed yellow line indicates the proposed extent of the SZ in this conceptual schematic; however, it does not denote a histologically demarcated boundary or an independently established tissue entity.

Figure 7 provides spatial context for the proposed TZ–SZ framework by contrasting the soft-tissue corridors associated with equicrestal and subcrestal implant placement; however, these clinical configurations do not determine the histologic composition of either region.

The apparent continuity observed in Case B is compatible with the TZ and SZ representing spatially differentiated regions along a continuum within the same peri-implant soft-tissue environment rather than separate anatomic structures [3,4,5,6,7,8,9,10,11,26,27]. Their relationship may therefore be better understood as a tissue gradient, with a conceptual rather than histologically discrete boundary.

In Case C, radial structural differentiation was observed between the metal-facing and bone-facing aspects of the SZ-compatible tissue.

Cases A and C underwent closed healing with a staged surgical approach, whereas Case B underwent open transmucosal healing with a one-stage approach. Case B also differed in anatomical site, abutment configuration and timing, and specimen composition; only its specimen contained both coronal and deeper tissue within a single sample. Therefore, the observed differences in epithelial distribution and connective tissue architecture cannot be attributed to healing mode. These findings remain descriptive, and the potential influence of healing conditions requires evaluation in standardized comparative studies.

Although functional properties were not evaluated, the observed tissue architecture permits a limited hypothesis regarding the potential interface-related role of the TZ–SZ complex. The epithelialized coronal portion of the TZ resembles the established peri-implant epithelial barrier [4,9,10,11], while its connective tissue component forms part of the peri-implant wound-healing and integration environment [28,29]. At a deeper level, the circumferential configurations observed in Cases A and C and the compact collagen organization on the metal-facing aspect of Case C raise the hypothesis that connective tissue adjacent to subcrestal healing components may contribute to an interface-related function [26,27]. However, these morphologic observations do not demonstrate sealing, protective, or mechanical stabilization functions, and such roles require direct experimental validation.

The present observations may have implications for the interpretation of peri-implant soft tissue around selected SPIs. Subcrestal placement may be used to accommodate ridge morphology and facilitate an appropriate restorative emergence profile [15,16,17,18,19,30,31,32]. The organized SZ-compatible connective tissue observed in these cases was characterized descriptively; the present findings do not establish whether this tissue is pathological or non-pathological. Unlike the first author’s previous conceptual and clinical reports [30,31,32], which did not include the histologic specimens or analyses presented here, the present study provides previously unpublished histologic observations from three human peri-implant soft-tissue specimens.

The long-term clinical and radiographic observations shown in Figure 3G–J provide patient-level context indicating that other SPIs in the same patient as Case B remained in function at the available follow-up examinations. They do not, however, establish the long-term persistence or histologic identity of tissue occupying the SZ-compatible compartment. Overall, the proposed SZ framework provides a spatial basis for interpreting organized connective tissue adjacent to subcrestal implant components [3,4,5,6,7,8,9,10,11,12,13,14,15,16,17,18,33,34].

## 4. Limitations

A central limitation of this report is the clinically selected nature of the specimens. In all three cases, tissue was retrieved because it interfered with a surgical or prosthetic procedure and therefore required removal as part of treatment. The specimens consequently represent prominent, treatment-interfering tissues rather than randomly sampled, protocol-driven, or longitudinally stable peri-implant tissues. Accordingly, they should not be interpreted as demonstrating that the same histologic organization routinely occurs around stable SPIs or as being representative of the broader SPI population. This sampling constraint was unavoidable because removal of clinically stable peri-implant tissue solely for research purposes would not have been ethically justified. The present findings should therefore be regarded as descriptive and hypothesis-generating.

For Cases A and C, postoperative follow-up was descriptive rather than a prospectively standardized peri-implant health assessment.

Additional limitations include the small number of specimens and differences in implant site, healing condition, specimen geometry, tissue retrieval, orientation, and sectioning strategy. In Case B, the clinically required tissue removal resulted from the dimensional and geometric difference between the digital abutment used during healing and the definitive restoration, as described above. The analyses were limited to the available histologic sections and did not include quantitative histomorphometry, immunohistochemical or microbiologic assessment, polarized-light evaluation or quantification, or ultrastructural evaluation. In particular, the absence of microbiologic testing precludes conclusions regarding microbial conditions in open and closed healing environments. Histologic correlation with long-term tissue persistence was not possible. Larger comparative studies using standardized sampling and analytic protocols are required to determine the reproducibility and biologic significance of the observed subcrestal connective tissue organization.

## 5. Conclusions

This three-case histologic report identified distinct patterns of connective tissue organization within subcrestal peri-implant compartments. Case A demonstrated a circumferential connective tissue configuration, Case B showed apparent continuity between a coronal epithelial–connective tissue region and a deeper collagen-rich compartment, and Case C demonstrated structural differentiation between the metal-facing and bone-facing aspects of an SZ-compatible specimen.

These observations demonstrate that organized collagen-rich connective tissue can be present within the subcrestal peri-implant environment and is compatible with the proposed SZ framework. However, the findings do not establish the SZ as a distinct tissue type or demonstrate its long-term persistence, biologic function, or clinical benefit. Standardized comparative and longitudinal studies are required to clarify the biologic identity and clinical significance of SZ-compatible tissue.

## Data Availability

The datasets used and analyzed during the current study are available from the corresponding author upon reasonable request.

## Supplementary Materials

The following supporting information can be downloaded at https://www.mdpi.com/article/10.3390/jcm15207807/s1, CARE Checklist.

## Abbreviations

CBCT	Cone-beam computed tomography
EPI	Equicrestally placed implant
H&E	Hematoxylin and eosin
SPI	Subcrestally placed implant
STA	Supracrestal tissue attachment
SZ	Subcrestal Zone
TZ	Transitional Zone
