# Supplementary material for: Histologic Patterns of Connective Tissue Organization in the Subcrestal Peri-Implant Compartment: A Three-Case Report
Source: J Clin Med. 2026 Oct 10;15(20):7807. doi: 10.3390/jcm15207807 (PMC13705394; doi:10.3390/jcm15207807)
Supplement: Supplementary file 1 [file jcm-15-07807-s001.zip › jcm-4588239-supplementary.pdf]

CARE Checklist

Completed against the 2013 CARE case-report reporting guideline

**Manuscript ID**	jcm-4588239
**Title**	Histologic Patterns of Connective Tissue Organization in the Subcrestal Peri-Implant Compartment: A Three-Case Report
**Authors**	Chiyun Won and Mi Heon Ryu
**Manuscript assessed**	Won and Ryu version 45.1 for JCM (36-page PDF with continuous line numbering)

Topic	Item	CARE requirement	Status	Reported location / comment
**Title**	**1**	Primary focus followed by the words 'case report'.	**Reported**	p. 1, lines 1-2. Title ends with 'A Three-Case Report'.
**Key words**	**2**	Keywords identifying the main focus, including 'case report'.	**Reported**	p. 2, lines 38-39. The keyword 'case report' is included.
**Abstract**	**3a**	Why the cases are of interest and what they add.	**Reported**	p. 2, lines 22-26 and 34-37.
**Abstract**	**3b**	Main concerns or important clinical findings.	**Reported**	p. 2, lines 27-33 (principal histologic findings across Cases A-C).
**Abstract**	**3c**	Main diagnoses, interventions, and outcomes.	**Reported**	p. 2, lines 27-37.
**Abstract**	**3d**	Principal take-away lesson.	**Reported**	p. 2, lines 34-37.
**Introduction**	**4**	Brief explanation of novelty and scientific context.	**Reported**	pp. 3-4, lines 41-77.
**Patient information**	**5a**	De-identified patient-specific information.	**Reported**	Case A: pp. 8-9, lines 145-164; Case B: pp. 13-14, lines 233-258; Case C: pp. 18-19, lines 314-333.
**Patient information**	**5b**	Primary concerns and symptoms.	**Reported**	Case A: lines 145-148; Case B: lines 233-236; Case C: lines 314-317.
**Patient information**	**5c**	Relevant medical, family, psychosocial, and genetic history.	**Not reported**	No relevant history is reported; the manuscript is focused on retrieved peri-implant soft-tissue specimens.
**Patient information**	**5d**	Relevant previous interventions and outcomes.	**Reported**	Case A: lines 145-164; Case B: lines 233-258; Case C: lines 314-330.
**Clinical findings**	**6**	Important examination and clinical findings.	**Reported**	Case A: pp. 7-11; Case B: pp. 11-16; Case C: pp. 17-20, including figure legends and case narratives.
**Timeline**	**7**	Historical and current information organized chronologically.	**Partial**	Chronology is reported narratively: Case A lines 145-164; Case B lines 233-252; Case C lines 314-330. No separate timeline table or figure is provided.
**Diagnostic assessment**	**8a**	Diagnostic methods, including examination and imaging.	**Reported**	Clinical examination and CBCT/panoramic/intraoral imaging: pp. 7-20, especially lines 128-164, 196-252, and 299-330.
**Diagnostic assessment**	**8b**	Diagnostic challenges.	**Not applicable**	No material diagnostic access, financial, cultural, or comparable challenge was identified.
**Diagnostic assessment**	**8c**	Diagnosis and relevant alternatives considered.	**Reported**	Clinical diagnoses/conditions: lines 145-155, 233-243, and 314-322. Alternative interpretation of the retrieved tissue is discussed at pp. 25-29, lines 447-517.

Topic	Item	CARE requirement	Status	Reported location / comment
**Diagnostic assessment**	**8d**	Prognostic characteristics, when applicable.	**Not applicable**	No disease staging or comparable prognostic classification was applicable to this descriptive histologic report.
**Therapeutic intervention**	**9a**	Type of intervention.	**Reported**	Extraction, socket preservation, implant placement, healing approach, second-stage/prosthetic procedures, and tissue retrieval: pp. 8-20.
**Therapeutic intervention**	**9b**	How the intervention was administered, including relevant dimensions and duration.	**Reported**	Device dimensions, procedure dates, and healing intervals: lines 145-164, 233-252, and 314-330.
**Therapeutic intervention**	**9c**	Changes to intervention and rationale.	**Reported**	Case A bone/soft-tissue adjustment: lines 156-164; Case B restoration adjustment and remake: lines 244-252; Case C tissue removal for seating: lines 323-330.
**Follow-up and outcomes**	**10a**	Clinician- and patient-assessed outcomes, if available.	**Reported**	Case A lines 162-164; Case B lines 249-252; Case C lines 328-330.
**Follow-up and outcomes**	**10b**	Important follow-up tests and diagnostic results.	**Reported**	Figure 1 and Figure 3; lines 139-143 and 214-231; clinical follow-up in lines 162-164, 249-252, and 328-330.
**Follow-up and outcomes**	**10c**	Adherence and tolerability.	**Partial**	No formal adherence measure was used. Clinical tolerance/outcome is described; Case B reports no food impaction (lines 249-252).
**Follow-up and outcomes**	**10d**	Adverse or unanticipated events.	**Reported**	Healing was uneventful in Cases A and B (lines 153-155 and 244-248); no clinical inflammation or recession at Case C follow-up (lines 328-330).
**Discussion**	**11a**	Strengths and limitations.	**Reported**	Discussion pp. 25-29, lines 439-517; Limitations pp. 29-30, lines 519-534.
**Discussion**	**11b**	Relevant literature.	**Reported**	pp. 25-29, lines 439-517, with references.
**Discussion**	**11c**	Scientific rationale for the conclusions.	**Reported**	pp. 25-29, lines 454-517.
**Discussion**	**11d**	One-paragraph take-away conclusion without references.	**Reported**	p. 30, lines 536-546.
**Patient perspective**	**12**	Patient perspective on treatment.	**Not reported**	A patient-perspective paragraph is not included.
**Informed consent**	**13**	Confirmation of informed consent.	**Reported**	p. 32, lines 581-583. Written informed consent was obtained from all patients for publication of clinical and histologic materials and images.

Checklist completion note

The clinical chronology is reported within the three case narratives and is mapped to the relevant line numbers under item 7. Patient perspective was not systematically collected and is therefore identified as not reported under item 12. Items that were not applicable to this descriptive histologic case report are labeled accordingly.

Guideline source: CARE Case Report Guidelines, 2013 CARE Checklist (care-statement.org). Locations were verified against the authors' final 36-page PDF.
